# Incorporating Diurnal and Meter-Scale Variations of Ambient CO_2_ Concentrations in Development of Direct Air Capture Technologies

**DOI:** 10.1021/acssuschemeng.4c06158

**Published:** 2024-10-25

**Authors:** Shubham Jamdade, Xuqing Cai, Melissa R. Allen-Dumas, David S. Sholl

**Affiliations:** † School of Chemical & Biomolecular Engineering, Georgia Institute of Technology, Atlanta, Georgia 30332-0100, United States; ‡ Oak Ridge National Laboratory, Oak Ridge, Tennessee 37830, United States

**Keywords:** direct air capture, carbon dioxide removal, eddy flux measurements, atmospheric conditions, facility siting

## Abstract

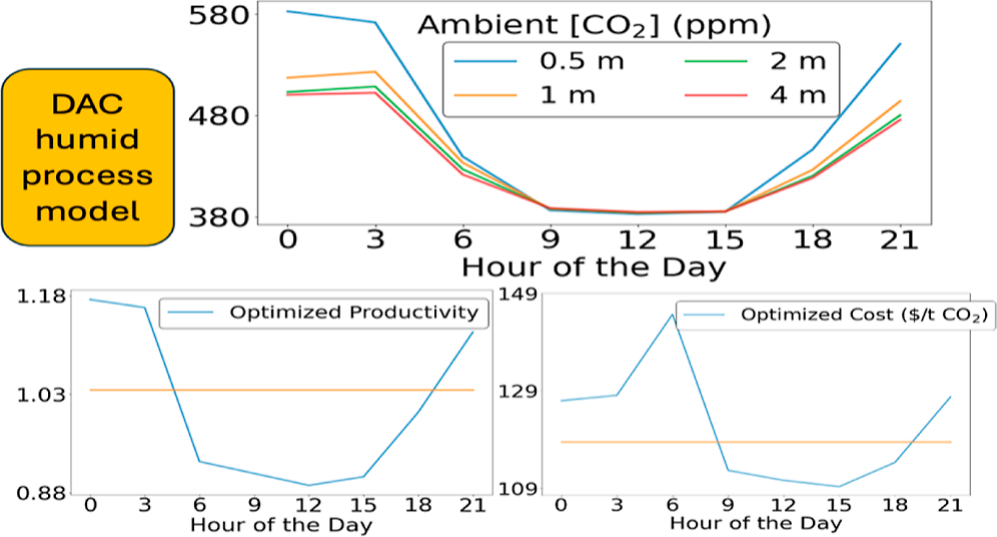

To be implemented on climate-relevant scales, direct air capture of CO_2_ (DAC) will require large capital-intensive facilities and careful attention to cost minimization. In making decisions among potential sites for DAC facilities, all of the factors that will impact process cost and efficiency should be considered. In this paper we focus on a factor that has previously received little attention in the DAC community, namely variations in atmospheric conditions on hourly time scales and length scales of meters. We present data curated from extensive previous studies of biosphere-atmosphere fluxes with observations of CO_2_ concentration, temperature, and relative humidity (RH) with hourly resolution from many sites in North America. These include locations where typical diurnal variations in CO_2_ concentration during summer months exceeds 150 ppm. These variations are larger than the seasonal variations that exist between averaged CO_2_ concentrations in winter and summer, and they are highly correlated with diurnal variations in temperature and RH. Diurnal variations are dependent on the height above ground at which CO_2_ concentrations are measured, with smaller variations existing at heights of 10 m or more than at ground level. We illustrate the potential implications of these short-term variations for the operation and optimization of a DAC process with process-level calculations for a specific adsorption-based process using amine-rich adsorbents.

## Abstract 2

Strong variations in ambient CO_2_ concentrations on hourly time scales have important implications for efficient direct air capture of CO_2_.

## Introduction

For direct air capture of CO_2_ (DAC) to be implemented at climate-relevant scales, large installations that operate in fixed locations must be envisaged. This situation is analogous in many ways to the chemical process industry, where large scale plants often require capital investments of $1B or more and operation over decades is assumed for the purposes of financial costing.^1−3^ Selecting sites for these kinds of large and enduring investments should fully consider the variations and trade-offs that exist between possible sites. An inescapable source of variation between geographically distinct DAC sites is the properties of DAC’s “feedstock”, i.e. air. For example, the annual average CO_2_ concentration within the contiguous United States varied by ∼5%, from 409 to 431 ppm, in 2018.^4^ If the available CO_2_ is expressed volumetrically (e.g., g CO_2_/m^3^ air), this annual variation is >40%, with most of this variation occurring because of differences in air pressure with elevation.^5^

Recent work by Cai et al. emphasized that the considerable variations in CO_2_ availability from ambient air that exist on time scales of months have important implications for modeling the efficiency of DAC processes.^5^ These variations are well-known from the famous Keeling curve.^6^ These variations are strongly correlated with seasonal shifts in average temperature and humidity, which also often create substantial changes in the efficiency and energy use of DAC processes. Cai et al. showed for a specific adsorption-based DAC process that including information about monthly averages of CO_2_ concentrations and meteorological conditions led to different choices about optimal process parameters and siting than models based simply on nationally averaged conditions.^5^ Cai et al. focused on DAC in the continental United States. Similar effects have been noted by Schellevis et al. based on meteorological data from The Netherlands.^7^ An implication of this work was that direct measurements of DAC process performance under a representative set of atmospheric conditions (CO_2_ concentrations, temperatures, humidities) should be made before geographically distinct DAC sites can be carefully compared.

The work by Cai et al. considered temporal variations of months and spatial variations on scales of hundreds of kilometers. In this paper, we consider much smaller scales, namely temporal scales of hours and spatial scales of meters. A large number of careful measurements of the properties of CO_2_ in air have been made on these scales by the research community using eddy flux measurements to quantify net biosphere-atmosphere fluxes.^8^ For example, the FLUXNET2015 project has compiled more than 1500 site-years of hourly CO_2_ and meteorological data from more than 200 sites around the world.^9^ As we show below, these measurements can have enormous value for making operational and siting decisions for DAC processes. We also highlight how longstanding choices made by the eddy flux research community may leave key data gaps from the perspective of DAC processes, pointing to the need for focused field experiments in developing national and regional strategies to enable DAC.

## Results

### Diurnal Variations in CO_2_ in Ambient Air

**Figure 1:**
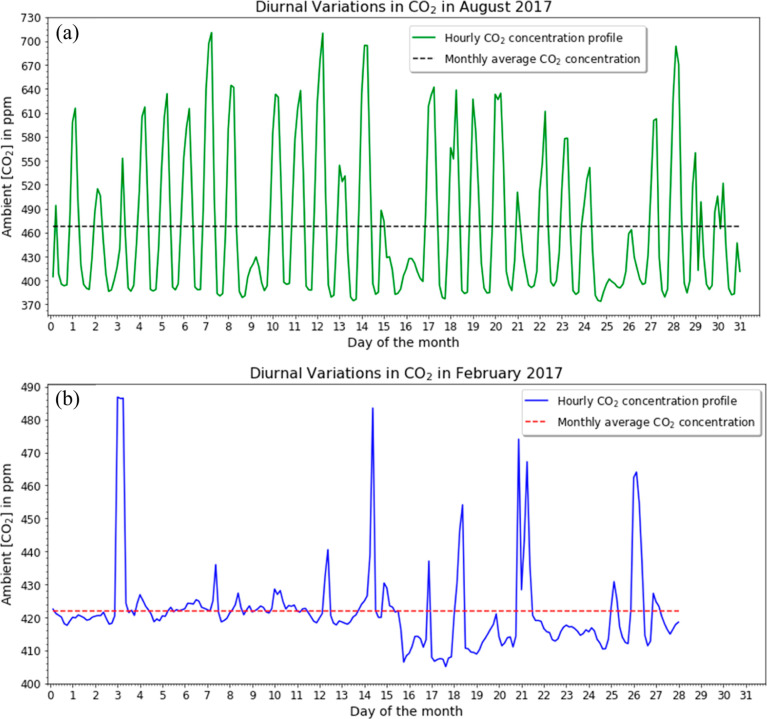
Ambient CO_2_ concentrations profile at a height of 0.5 m from SPRUCE site 07 from (a) August 01–August 31, 2017, and (b) February 01–February 28, 2017. The solid line shows average CO_2_ concentrations over 3 h periods for all days in a month, while dashed lines indicate the monthly averaged concentrations. Minor ticks on the horizontal axes are at 3 h intervals.

During seasons when plants in a local environment are growing, CO_2_ flux into and out of plants from the atmosphere occurs by CO_2_ uptake during photosynthesis and CO_2_ release during respiration.^8^ Photosynthesis only occurs during the day, but so-called “dark” respiration occurs at all times, leading to local ambient CO_2_ concentrations that are lower than average during the day and higher than average at night. It may be surprising to many researchers working on DAC how strong these variations can be. An example of field measurements from the Oak Ridge National Laboratory’s SPRUCE experiments^10^ is shown in Figure 1. The SPRUCE experiments took place in a peat bog in Minnesota and included enclosed canopies in which tests with CO_2_ injection and/or ground heating were performed. The data in Figure 1 is from a control site that had no containing canopy or external forcing, that is, data for ambient air. Figure 1 is a good example of the variations in average CO_2_ concentrations on seasonal scales discussed above; the monthly averaged CO_2_ concentration in August and February 2017 was 467 and 422 ppm, respectively.

We quantified the daily fluctuations in this data by considering the difference in average CO_2_ concentration between midnight and noon. During August 2017 at the SPRUCE site there were 8 days where the diurnal fluctuation was between 0 and 100 ppm, 10 days where it was between 100 and 200 ppm, and 13 days where the range was greater than 200 ppm. The highest observed diurnal variation was 318 ppm. In this month of data there were 16 days during which the peak CO_2_ concentrations were greater than 600 ppm; the highest recorded concentration was 747 ppm and the lowest recorded concentration was 372 ppm. A striking observation from Figure 1 is that the variations of CO_2_ concentrations on hourly scales are far larger than the seasonally averaged variations across the US analyzed by Cai et al. (409–431 ppm in 2018).^5^

**Figure 2:**
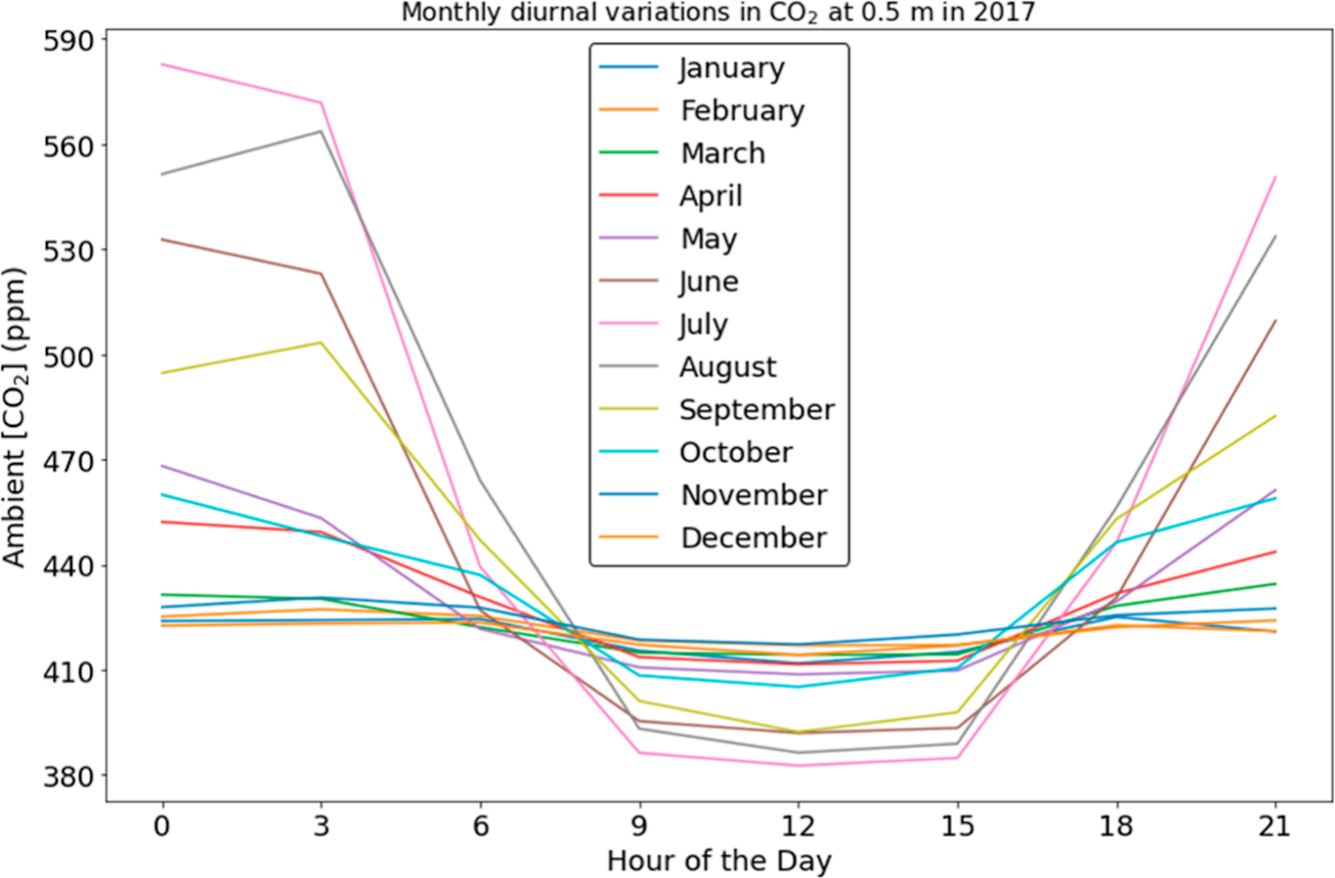
Average ambient CO_2_ concentrations at a height of 0.5 m from SPRUCE site 07 plotted at 3 h intervals for all months in 2017. The CO_2_ concentration value at each specified hour is the average concentration over a 3 h period starting from that hour averaged across all days of the month.

The diurnal patterns associated with the CO_2_ concentrations on hourly scales at the SPRUCE site are shown in Figure 2, which shows the average concentration at specific times during the day averaged over all observations in a month. In summer months, such as the August data highlighted in Figure 1a, the peak concentration of CO_2_ occurs after midnight and CO_2_ concentrations drop rapidly after sunrise. In the August data in Figure 1a, the monthly average CO_2_ concentration at 6 AM was 516 ppm and at 9 AM it was 400 ppm. As outlined above, this diurnal pattern exists because photosynthesis only takes place when sunlight is present, but plant respiration occurs in the day and night.^8^

Because of the biogenic origin of the diurnal variations in the summer data in Figures 1 and 2, it is not surprising that the diurnal variations in CO_2_ concentration are much smaller during winter, when plant growth in locations such as the SPRUCE site is minimal. As shown in Figure 1b for February 2017, 23 days had diurnal fluctuations in CO_2_ concentrations less than 10 ppm, 4 days showed a range of 10 to 50 ppm and 1 day had a range of 114 ppm. In February 2017, the highest recorded CO_2_ concentration was 535 ppm and the lowest recorded CO_2_ concentration was 405 ppm.

Although the observations discussed above may not be widely known in the DAC community, the diurnal swings in CO_2_ concentration illustrated in Figure 1 will be unsurprising to researchers who have studied biosphere-atmosphere fluxes. For example, similar effects have been reported in measurements in a subalpine forest in Colorado by Jarvis et al.^11^ and Bowling et al.^12^ In measurements close to ground level, Jarvis et al. reported daily variations in CO_2_ concentration during summer of ∼80 ppm. Because these swings have biogenic origins, it should not be surprising that different land and vegetation types lead to different levels of variability in CO_2_ concentrations. The SPRUCE data in Figure 1 and the subalpine forest data of Jarvis et al. is an example indicating that the peat bog location of the SPRUCE experiments has a more active summer respiratory cycle than subalpine forests. We return to this issue in a more general way below by examining observational data from a range of sites across the US.

The SPRUCE site and the subalpine forest used by Jarvis et al. also provide a useful illustration of the significant role of atmospheric pressure on the net availability of CO_2_ from air. Treating air as an ideal gas, the mass of CO_2_ in kg in 1000 m^3^ of air is 1.958 × *C*_CO2_ × *P* × (273/*T*), where *C*_CO2_ is the CO_2_ concentration in ppm, *P* is air pressure in atm, and *T* is the air temperature in K. At a constant concentration of 420 ppm of CO_2_ and 25 °C, 1000 m^3^ of air at the SPRUCE site (elevation 418 m) contains 753 kg of CO_2_, while the same volume of air at the Colorado site (elevation 3050 m) contains 529 kg of CO_2_.

### Correlation Between Hourly CO_2_ Concentration and *T* and RH

Although the concentration of CO_2_ in air is obviously important for the efficiency and optimization of DAC processes, other properties of ambient air can also play a significant role.^5,7^ In adsorption-based processes, for example, increased humidity can enhance the efficiency of CO_2_ capture but also increase the energy requirements for CO_2_ release from an adsorbent. Similarly, lower ambient air temperatures can lead to stronger adsorption of CO_2_ in a capture material while potentially increasing costs associated with regeneration. Cai et al. used extensive process optimization to illustrate these issues for a prototypical adsorption-based DAC process.^5^ Because of the interplay between these factors in process optimization, it is important to recognize that the diurnal variations in CO_2_ concentration illustrated above are highly correlated with diurnal variations in temperature and relative humidity (RH).

**Figure 3:**
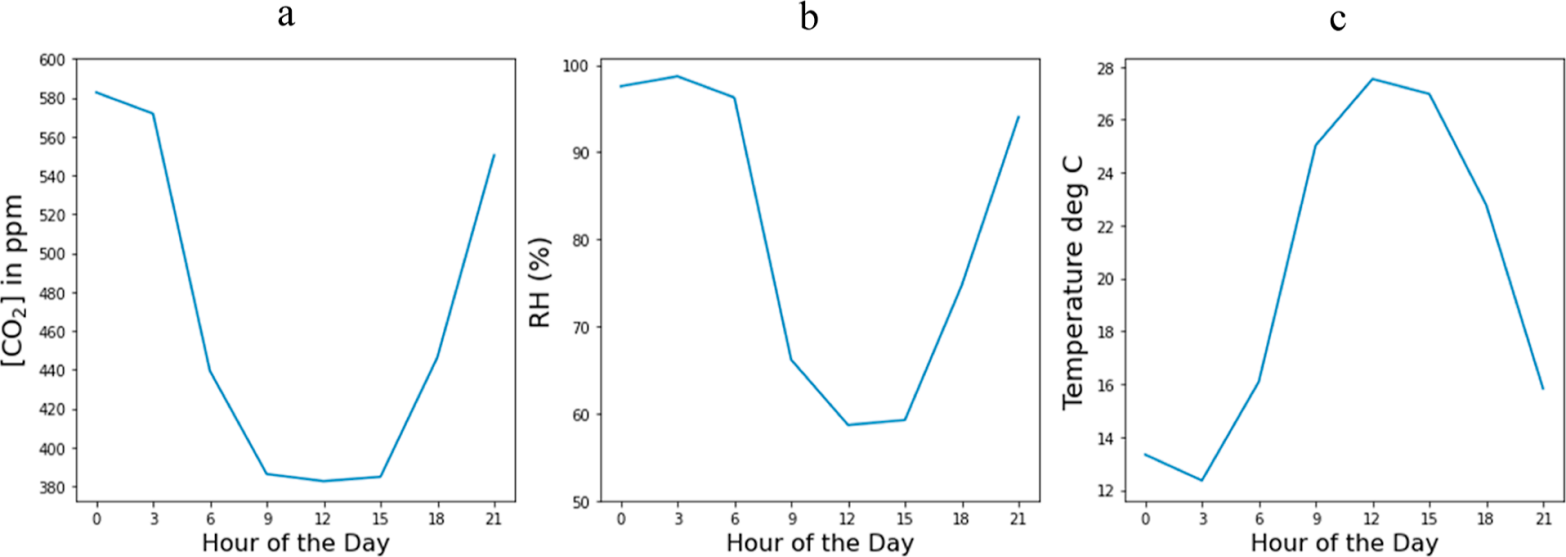
Average ambient (a) CO_2_ concentration, (b) RH and (c) temperature at a height of 0.5 m from SPRUCE site 07 plotted at 3 h intervals for July 2017. The value at each specified hour is the average concentration over a 3 h period starting from that hour, averaged across all days of the month.

The correlation between CO_2_ concentration, humidity, and temperature for the SPRUCE site in July 2017 is shown in Figure 3. For each quantity, this figure shows measurements for specific times of day averaged over all data from the month of July. The diurnal variation of RH (Figure 3b) displays a similar pattern to the CO_2_ concentration (Figure 3a). Warm air can hold more water vapor because the higher temperature results in increased thermal energy that tends to keep water molecules in the gaseous vapor phase before saturation and condensing into liquid water droplets.^13^ Unsurprisingly, the diurnal variation of temperature (Figure 3c) displays an opposite pattern to the CO_2_ concentration and RH.

### Variations in CO_2_ in Ambient Air on Vertical Scales of Meters

**Figure 4:**
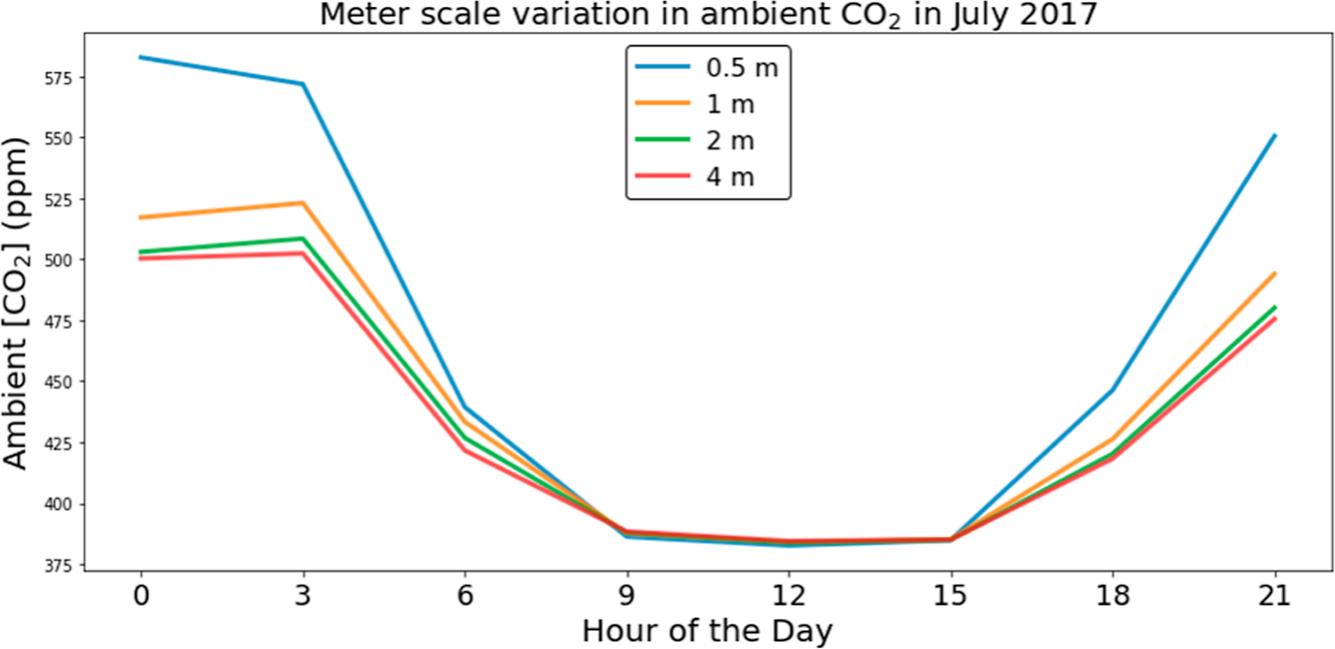
Average ambient CO_2_ concentrations at a height of 0.5, 1, 2, and 4 m from SPRUCE site 07 at 3 h intervals for July 2017. The CO_2_ concentration value at each specified hour is the average concentration over a 3 h period for that hour averaged across all days of the month.

The diurnal variations in CO_2_ concentrations seen in Figure 1a are best understood as showing net generation of CO_2_ during the night via respiration and a smaller consumption of CO_2_ during the day from a combination of respiration and photosynthesis. The CO_2_ generated during the night becomes dispersed over time into higher levels of the atmosphere, a process strongly influenced on local scales by boundary layer mixing.^8^ An implication of this situation that may become important for operating DAC processes is that there can be variation in CO_2_ concentrations at different heights above the ground. This vertical variation has been carefully considered in earlier measurements in the eddy-flux research community. Bowling et al., for example, demonstrated the relative influences of photosynthesis, respiration, and atmospheric dynamics on CO_2_ concentrations at canopy heights ranging from 0.1 to 21.5 m.^12^ They recorded considerable vertical variation in CO_2_ concentration during the night when vertical mixing is limited and smaller variations during the day when convective heating increases vertical mixing. Similar trends in CO_2_ concentration were observed for a more limited range of observation heights at the SPRUCE site, as illustrated in Figure 4. The average diurnal variation in July 2017 for the SPRUCE site shown in Figure 4 was 200 ppm for measurements at a height of 0.5 m, compared to 119 and 116 ppm at heights of 2 and 4 m. There is little variation with respect to measurement height for temperature or RH (see Figures S2 and S3). As might be expected from Figures 1 and 2, there is little vertical variation in CO_2_ concentrations during winter months (see Figure S1).

A primary motivation of many studies that have measured local CO_2_ concentrations has been to quantify net biosphere-atmosphere CO_2_ fluxes. In making these so-called eddy flux measurements, the variations in concentration that occur on length scales of meters near the ground are expected to confound the overall CO_2_ fluxes of interest. As a result, these field studies have often been performed using relatively tall towers with the aim of taking measurements outside the near-ground boundary layer.^14−16^ We examined data available from the 212 sites (89 sites in North America, 6 in Africa, 13 in Asia, 23 in Australia, 68 in Europe, 8 in Russia and 5 sites in South America) included in the FLUXNET2015 2015 data set.^9^ Of these 212 sites, tower height information is available for 206 sites. Among these 206 sites, 111 sites have towers with a height below 10 m, while the remaining 95 sites made measurements more than 10 m above ground/canopy level.^9^ It is unclear what measurement height or heights are most relevant to large-scale DAC installations, but it seems likely that air intakes for these installations will typically be less than 10 m above ground.

**Figure 5:**
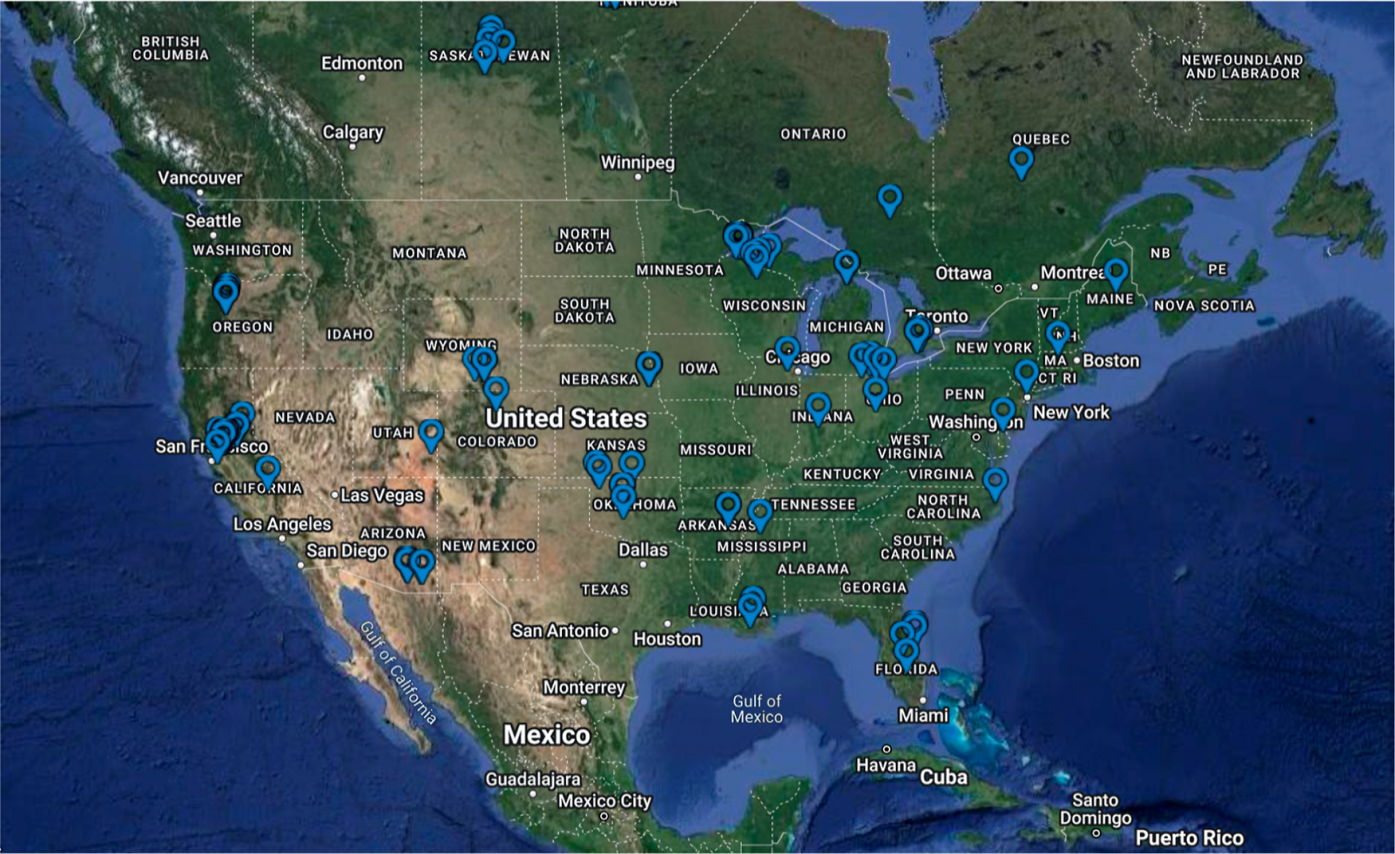
North America site locations reported in the FLUXNET2015 database,^9^ highlighting the prevalence of sites located in plant-rich areas (greener region). This map is sourced from the data portal that serves the FLUXNET2015 community at (https://fluxnet.org/sites/site-list-and-pages/?view=map). Reprinted with permission under a Creative Commons Attribution 4.0 International (CC BY 4.0) (https://creativecommons.org/licenses/by/4.0/) from ref (9). Copyright 2020 Nature/Scientific Data.

We have emphasized above the pivotal role of biomass in the diurnal and short length-scale variations that occur in ambient CO_2_ concentrations. Plant-rich locations have diverse range of vegetation types and growing seasons that lead to differences in ecosystem dynamics and carbon cycling processes.^17−20^Figure 5 illustrates the North America sites for which field measurements are collected in the FLUXNET2015 data set, showing that the majority of sites are situated in areas abundant with plants.^9^

**Figure 6:**
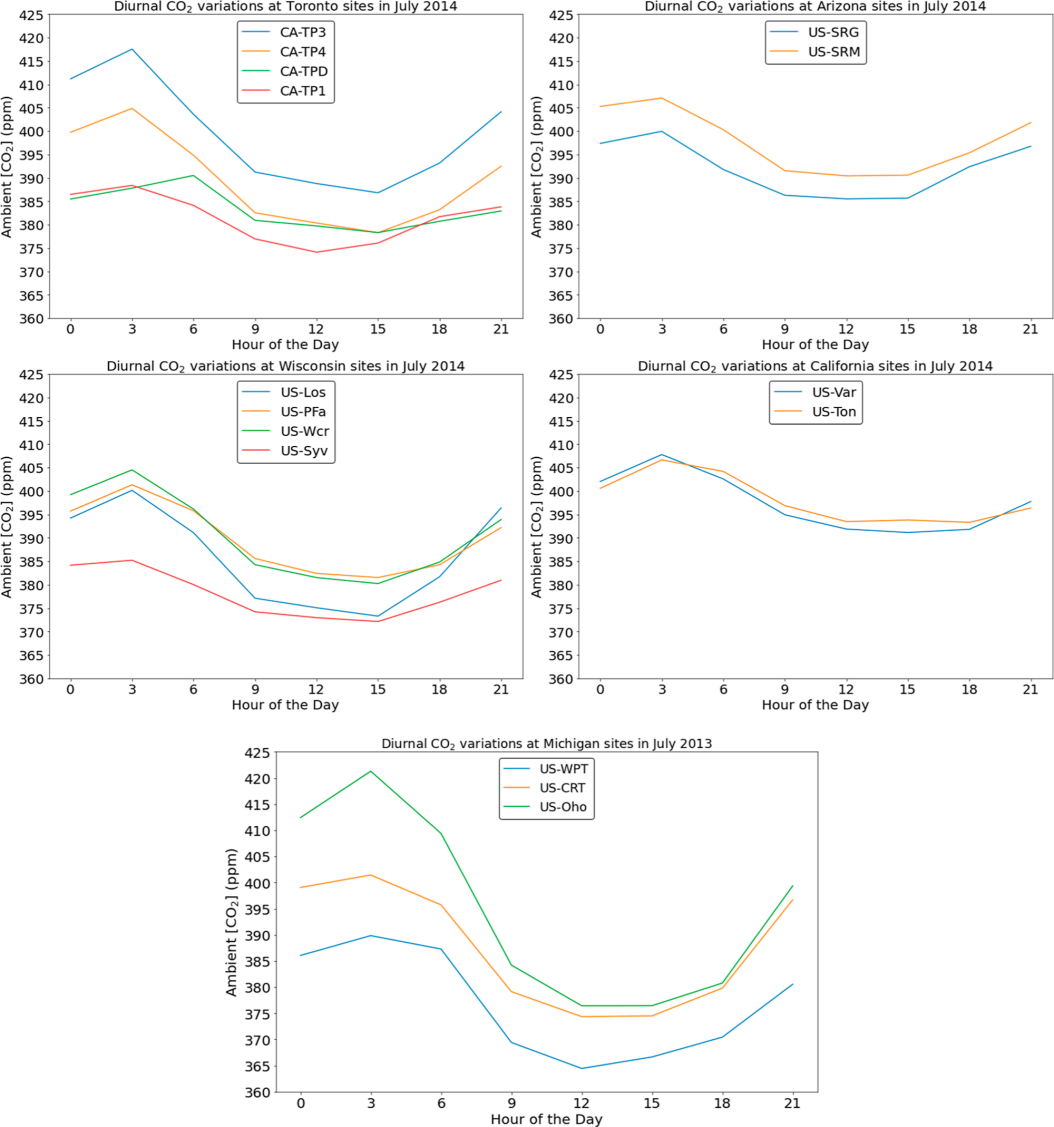
Average ambient CO_2_ concentration variation in the month of July at various regions across the US plotted at 3 h intervals. The legend in the plots represents the site ID. The CO_2_ concentration value at each specified hour is the average concentration over a 3 h period starting for that hour averaged across all days of the month. All data was obtained from field measurements reported in FLUXNET2015.

To enable an initial consideration of impacts of diurnal variability on DAC processes we selected several FLUXNET2015 sites from diverse climate zones in the North America, including Wisconsin (4 sites), Toronto (4 sites), Arizona (2 sites), Michigan (3 sites), and California (2 sites). We chose sites in each region that are in proximity with each other to allow comparative analysis within that region. We organized the FLUXNET2015 data from these sites in a format allowing ready access for researchers modeling DAC process modeling. Full data sets are provided in the Supporting Information, and a summary of the data for the month of July (chosen as representative of summer in the northern hemisphere) is shown in Figure 6. The full data sets include 170 years of data with CO_2_ concentration, RH, and temperature at hourly resolution. Table S1 summarizes the site details for above-mentioned 15 sites. Figure 6 shows the averaged diurnal variation of CO_2_ concentration in July at 15 sites. Sites within the specific regions follow similar CO_2_ variation trends. The small differences in CO_2_ concentration profiles among the sites in the same region may include variations in location elevation, tower height, temperature, and local vegetation density.

The diurnal variations observed at these sites are considerably smaller than the data from the SPRUCE data set shown in Figure 1. To probe the cause of this difference, we selected a site in Minnesota called US-MBP from the AmeriFlux Base database,^21^ which is in close proximity to the SPRUCE site. The FLUXNET2015 and AmeriFlux Base data sets share the same data source, with the difference that the AmeriFlux data set contains data up to 2022 and FLUXNET2015 data set includes data until 2014. An advantage of using the FLUXNET2015 data set is that a comprehensive data processing pipeline has been built on top of the AmeriFlux Base.^9,22^Figure S4 shows data from the US-MBP site in July 2017. The observed diurnal variation in CO_2_ concentration was 178 ppm, closely resembling the average diurnal variation of 200 ppm observed at the SPRUCE site. This comparison suggests that the variation in diurnal patterns between Figures 6 and 1 is a property of the geographical locations, not an artifact of data collection or similar factors.

We conducted an analysis of CO_2_ concentration variations measured at various tower heights for the subset of the 15 sites mentioned above for which this was possible (Figure S5). Tower heights and the ambient conditions data with respect to tower height were obtained from AmeriFlux Base database.^21^ The meter scale variations of CO_2_ concentration discussed in Figure 4 can also be detected at this diverse collection of sites, as shown in Figure S5. In almost all cases, higher tower heights led to lower diurnal variations than lower tower heights. The average diurnal variation in July decreased as the tower height increased and for most of the 24 h period, CO_2_ concentrations at lower tower heights were higher than those at taller towers.

### Implications of Hourly Variations in Ambient CO_2_ for a Prototypical Adsorption-Based DAC Process

The results above have highlighted how strong variations in CO_2_ concentration, humidity, and temperature can occur in ambient air. It is natural to ask how important these variations are to the efficient operation of DAC processes. A diverse array of processes have been proposed and tested for DAC,^23^ so it is not possible to give a comprehensive answer to this question addressing all possible DAC processes. Instead, we have performed process optimization calculations for a single well-defined DAC process to illustrate how these issues could be considered more broadly. To this end we used the same prototypical adsorption-based DAC process analyzed by Cai et al. in their study of impacts of seasonal and regional impacts of CO_2_ concentrations and air conditions.^5^ This process uses an amine-based adsorbent, and was selected because of extensive data that was available from Elfving et al.^24,25^ for the performance of the adsorbent as a function of temperature and RH. Adsorption-based processes with similar adsorbents have been reported and tested at a variety of scales.^26−31^

We performed calculations with the process model described in detail by Cai et al.,^5^ using total pressure, RH, CO_2_ concentration and temperature as process parameters. This process assumes adsorption occurs at ambient conditions and regeneration of the adsorbent is performed at a fixed temperature. We considered the same four performance metrics used by Cai et al.: productivity, recovery, electricity requirements and heat requirements. As in the work of Cai et al., we optimized adsorption and desorption times for the cyclic adsorption process for two separate scenarios, one in which DAC productivity was maximized (scenario 1) and a second in which DAC cost was minimized (scenario 2). Cai et al. performed this optimization assuming constant operating conditions in monthly time intervals. We applied the same model framework but considered much shorter time intervals to assess the impact of diurnal variations. Specifically, we used total pressure, RH, CO_2_ concentration and temperature values at eight points in a 24 h cycle (specifically, hours 0, 3, 6, 9, 12, 15, 18 and 21) that encompass the diurnal fluctuation. The ambient condition values at these points represent the average over a 3 h period starting from that hour, averaged across all days of the month, as shown in Figure 3. For every month, we used these eight ambient condition values to determine both maximized productivity or minimized cost at those points. For this analysis, we used recorded data from 2017 for SPRUCE site 07 with measurements taken at a height of 0.5 m. The SPRUCE data set did not have hourly total pressure data, so for all scenarios in this study we considered constant total pressure of 0.96 atm based on averaged data from a nearby weather station.^32^

**Figure 7:**
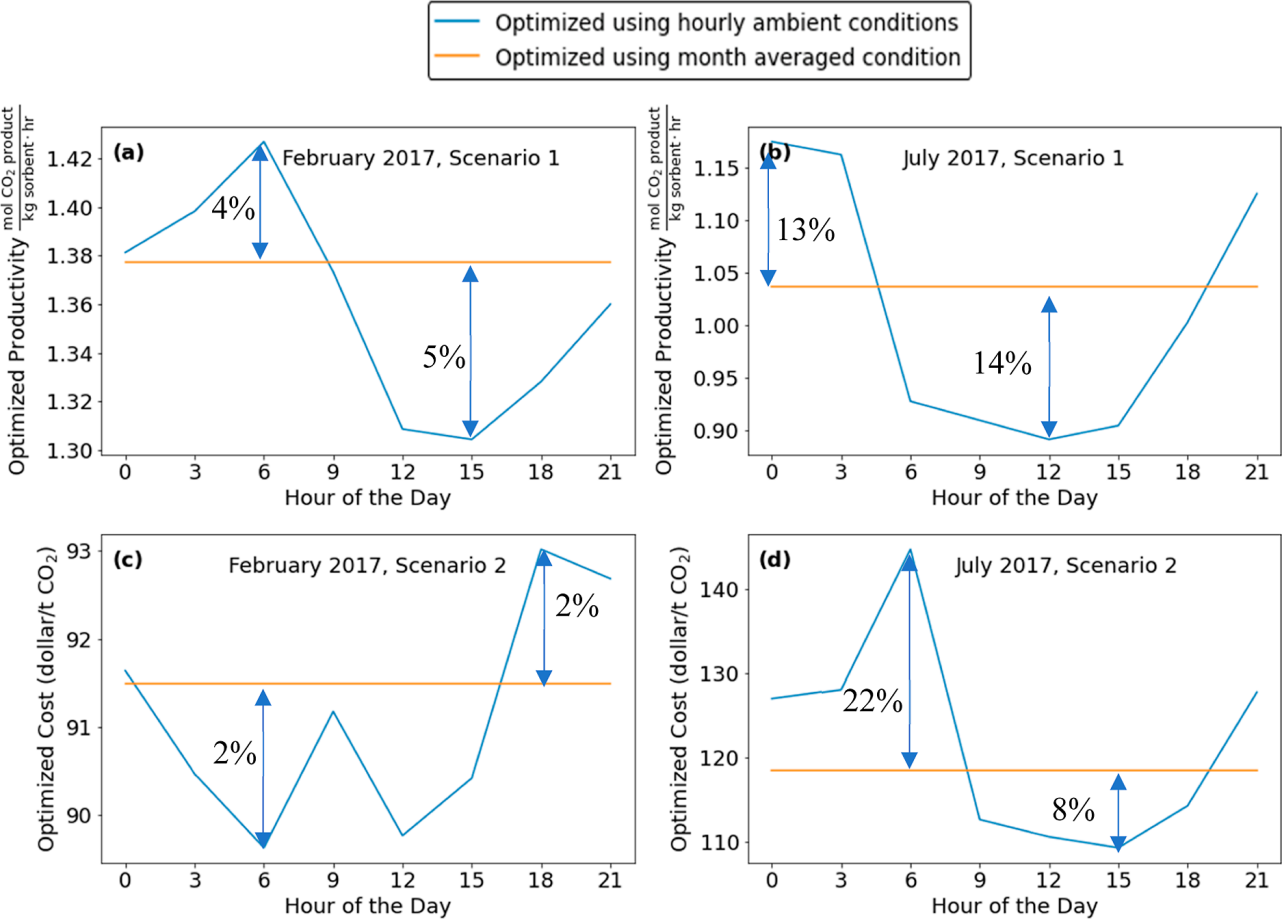
Optimization results for the adsorption-based DAC process described in the text showing performance optimized for productivity in (a) February 2017 and (b) July 2017 and performance optimized for cost in (c) February 2017 and (d) July 2017.

Figure 7a,b shows the variation in optimized productivity (scenario 1) throughout the day for the months of February and July, respectively. The hourly optimized productivity has a similar pattern to the diurnal variations of CO_2_ levels and RH, with higher productivity at night and lower productivity during the day. Larger variations in the productivity during a 24 h period are seen in summer than in winter. In July the nighttime productivity is more than 30% higher than the daytime productivity (Figure 7b), a difference that could have important implications for operating a large-scale facility. When optimization is performed to minimize cost (scenario 2), the hourly variations in cost are small in February (Figure 7c) but more substantial in July (Figure 7d). This is consistent with the observations of Cai et al. for this specific DAC process that ambient temperature and RH are negatively correlated to productivity and positively correlated to cost.^5^

A limitation of the cost estimates in the process model that we used is that the model assumes constant costs for electricity. In many industrial settings that cost of electricity varies on short time scales, including diurnal variations associated with overall support, demand, and the sources of electricity generation. For the specific process we considered, on average the electricity costs contributed 36 and 27% of the total optimized costs in Figure 7c,d, respectively. Although it is outside the scope of this work to incorporate these effects, understanding the sensitivity of process costs to these price variations will likely be important in detailed assessment of prospective DAC sites.

**Figure 8:**
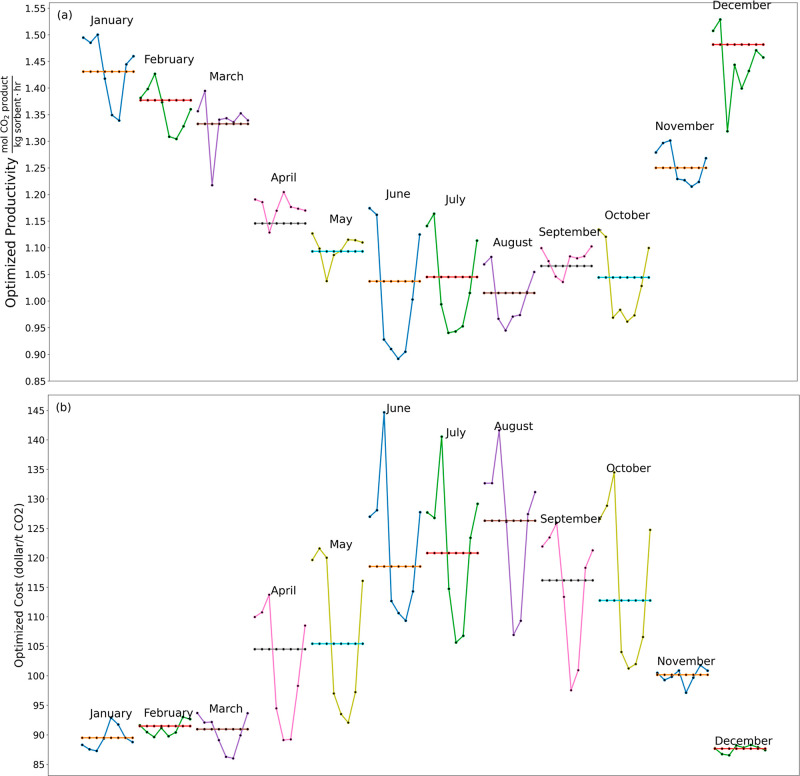
Optimization results for each month in 2017 at the SPRUCE site 07, presenting two scenarios: (a) performance optimized for productivity and (b) performance optimized for cost. The fluctuations within each month’s graph highlight typical daily trends, while the constant value line represents the optimized productivity/cost using month averaged conditions. The *x*-axis ticks corresponding to the markers in each graph represent the “Hour of the Day” with values “0, 3, 6, 9, 12, 15, 18, 21”.

Results analogous to Figure 7 for every month in the year at the SPRUCE site are shown in Figure 8. These results highlight the strong seasonal variations in DAC performance already identified by Cai et al. and the additional impacts of averaged diurnal oscillations in air conditions. The details of these results are specific to the adsorption-based DAC process we modeled, where the reductions in performance associated with increased humidity reduce the productivity increases that might be expected based on diurnal oscillations in CO_2_ concentration alone. If a DAC process was used for which productivity increases with RH it seems likely that the daily variations in performance associated with air conditions could be larger because of the strong correlation between CO_2_ concentration and humidity (Figure 3). The predicted total annual CO_2_ productivity from the two sets of calculations (i.e., optimization on an hourly basis and assuming constant conditions throughout a month) in Figure 8a differ by less than 1%. The predicted annual operating cost when including diurnal variation (Figure 8b), however is 6% lower than the estimate based on constant conditions in each month. This comparison is incomplete, however, because the process modeled as having constant conditions must actually be operated in an environment with diurnal variations in atmospheric conditions. We return to this point below.

**Figure 9:**
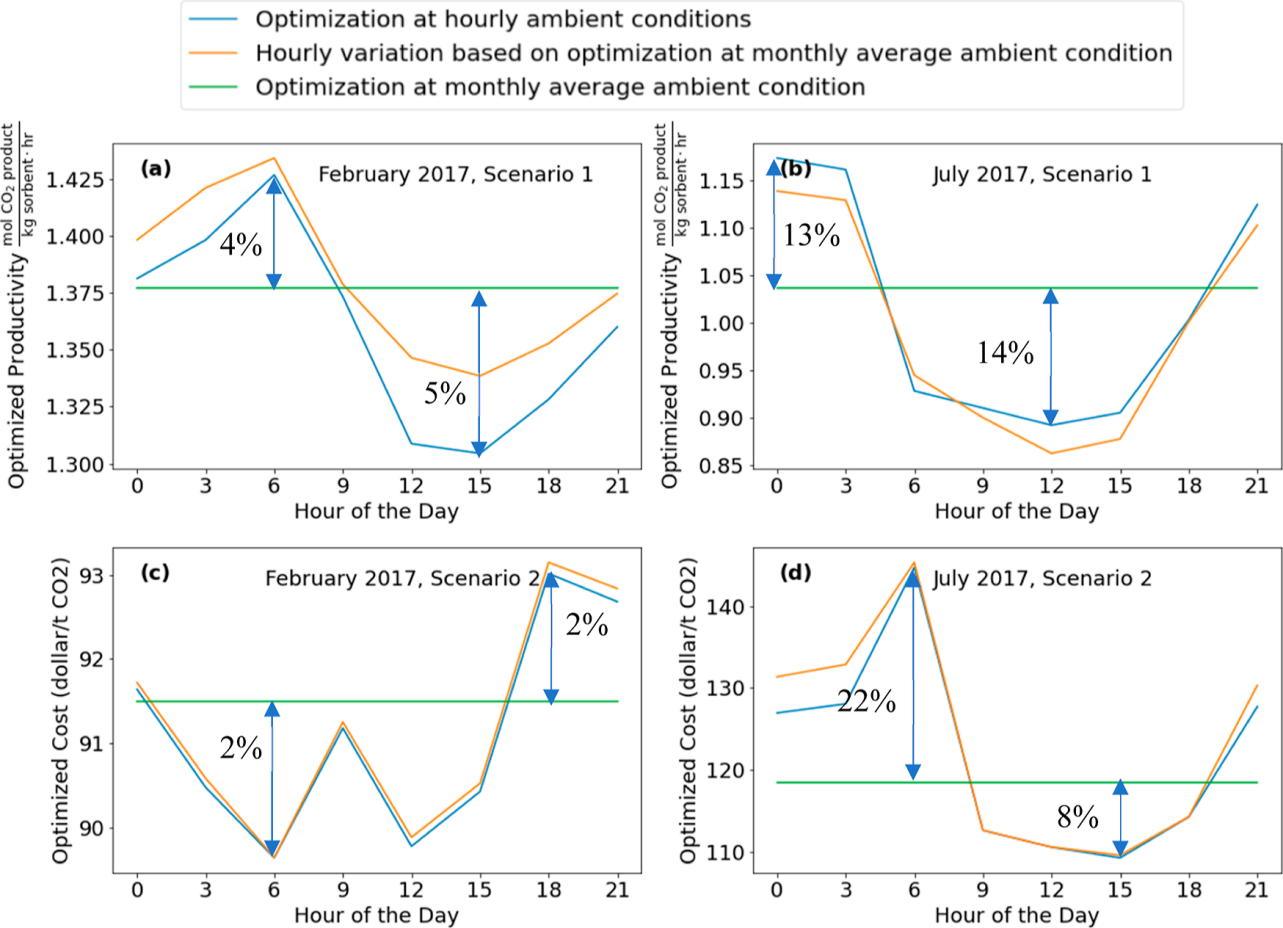
Optimization results for the adsorption-based DAC process described in the text showing performance optimized for productivity in (a) February 2017 and (b) July 2017 and performance optimized for cost in (c) February 2017 and (d) July 2017.

Figure 7 compared predicted DAC performance for the process we modeled assuming either constant conditions during a month or conditions varying at 3 h intervals. Results from these kinds of calculations could be used, for example, in comparing various possible geographic sites for a DAC facility.^5^ Even if the process conditions chosen from constant conditions are used, the actual process performance will differ systematically from the orange lines in Figure 7 because of diurnal variations. This systematic difference is shown for the months of February and July in Figure 9, where the orange curves show that actual performance of the DAC process operating at the constant process conditions but in an environment with diurnal variation in air conditions. Because the variations in process performance for the adsorption-based process we modeled as a function of the optimization variables (namely the adsorption and desorption times in each cycle) are not strong, the variations between the outcomes with constant process conditions during a month (orange curves) and the outcomes optimized at 3 h intervals (blue curves) are in many cases not large. Nevertheless, there are some instances where these differences are large enough that they may have practical implications for large-scale facilities. For example, operation aiming to minimize cost during July (Figure 9d) can reduce cost by ∼$4/t CO_2_ during the early morning. Given the US Department of Energy’s widely publicized goal of achieving DAC for a net cost of $100/t CO_2_, variations of even a few dollars per ton in cost will likely be important in the future. Ideally, the optimized productivity at 3 h intervals (blue curve in Figure 9a) should be higher than the hourly productivity variation observed under constant process conditions (orange curve in Figure 9a). The small deviation from this outcome observed in Figure 9a arose because of the tolerance thresholds used in our optimization formulation.

To assess the impact of diurnal variation on DAC performance we used 3 h averaged data as shown in Figures 2 and 4. This approach provided qualitative insights while minimizing the computational effort required for process optimization. As shown in Figure 1, however, actual hourly variation is more complicated than the averaged data. As operation of DAC facilities becomes more sophisticated there may be opportunities to further optimize performance by using real-time observational data or short time-horizon forecasts of similar information.

**Figure 10:**
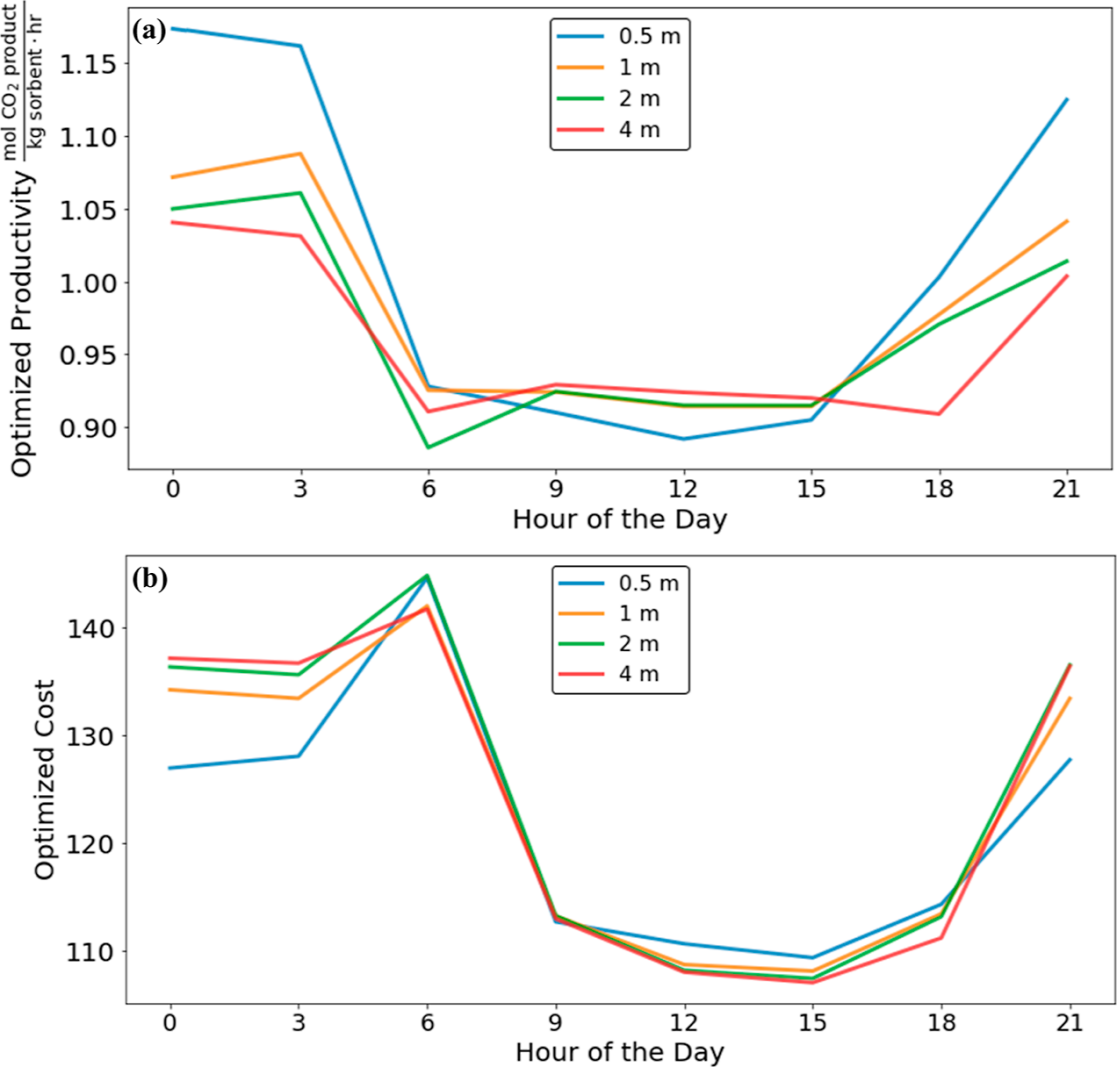
Variations in DAC performance for air intakes at various heights at SPRUCE site 07 using data from July 2017 (a) optimized for productivity (scenario 1) and (b) optimized for cost (scenario 2).

We showed above that significant variations in CO_2_ concentrations on a meter scale during nighttime (see Figure 4). To assess the potential impact of this phenomenon, we optimized the productivity (scenario 1) and cost (scenario 2) separately using ambient conditions at the SPRUCE site at heights of 0.5, 1, 2, and 4 m for the SPRUCE site. The results of these calculations are shown in Figure 10. As we noted above, temperature and RH show minimal variations with changes in height (see Figures S2 and S3). Thus, the differences in optimized productivity and costs using air from different heights are almost entirely due to the differences in CO_2_ concentration.

## Discussion and Outlook

For DAC to mature from a science-based pursuit focused on laboratory measurements in well-controlled settings to a climate-relevant sector characterized by large facilities at fixed locations it will be vital to consider the full range of factors that will affect performance in real-world settings. In this paper we have examined an issue that has not been widely appreciated in the DAC research community, namely the variations in CO_2_ concentration in ambient air that occur on hourly time scales and vertical length scales of meters. By leveraging large collections of field data we illustrated that at least in some locations these variations can be far larger than the seasonal variations that also exist. Because of the biogenic origins of diurnal variations in CO_2_ concentrations at the sites we considered, these diurnal variations are strongly correlated with similar variations in humidity and temperature.

We examined the potential implications of hourly and vertical variations in air conditions on the performance of a specific adsorption-based DAC process. Although estimations of process performance based on air conditions assumed to be constant during each month gave useful insight into this process, more careful optimization using diurnal variations allows noticeable improvements in the process productivity or cost. These improvements could have important implications when trying to fully optimize the performance of large-scale DAC installations. It is not reasonable to assume that the impacts of these hourly variations on all potential DAC processes will be similar to the specific process we analyzed. In the specific adsorption-based process we modeled, increased ambient humidity is correlated with lower performance, so the potential advantages of the higher CO_2_ concentration observed at nighttime are limited by the concomitant increases in humidity at these times. Processes whose performance is either neutral with respect to humidity or where performance is enhanced by higher humidity may be able to achieve advantages from diurnal variations that were not evident in the specific process we considered.

Our results suggest several recommendations for researchers and developers seeking to advance DAC. At the laboratory scale, the impacts of seasonal and diurnal variations in air conditions can only be assessed if measurements are made over a relevant range of temperature and humidity conditions. Information of this kind is vital if process-level modeling is to be used to impact of variations in air conditions on siting and performance of DAC facilities. The seasonal data compiled by Cai et al.^5^ and the examples of diurnal variations we have provided in this paper give insight into the envelope of conditions that need to be studied. As noted by Cai et al., there may be considerable value in the research community adopting a common set of test conditions to allow direct comparisons between independent measurements of the many possible processes that exist for DAC. Even in the absence of detailed parametric laboratory measurements, insight into the sensitivity of process performance on ambient air temperature, humidity, and CO_2_ concentration will be useful for understanding what issues are most important for future optimization of specific processes.

For developers of DAC facilities making choices about where to site facilities, a key conclusion from our work is that performing detailed measurements of air conditions over annual periods at a full range of prospective sites is strongly advisable. The data we have curated in this paper, which is based on impressive efforts over many years by the eddy flux community, can be used to perform initial assessments, but it suffers from shortcomings. Almost all of the data we reported is from sites that were deliberately chosen to be far from local anthropogenic CO_2_ sources (e.g., cities), and many of the sites made measurements only at a single height relatively far from ground level. It is not clear that data from, for example, heavily forested sites is fully representative of the local conditions that would be relevant for a large-scale DAC installation that might have an area of multiple hectares. Detailed measurements including data recorded as a function of height above ground level at specific potential DAC sites that broadens the range of sites that is already available from the sources we have used will have considerable value. The Hestia Project by Gurney et al.^33,34^ has begun to provide inventories of CO_2_ concentrations for individual cities with high spatial and temporal resolution. As further data of this kind becomes available the approach we followed in this paper could be extended to such data sets to gain a comprehensive understanding of CO_2_ variations in urban areas and their effect on DAC performance. Developing a strategy to collect data of this kind should be carefully considered in developing regional or national strategies to implement DAC at climate-relevant scales.

## Data Availability

The ZIP file with all the data files is available at this link. The ZIP file includes hourly atmospheric condition data from SPRUCE site 07, FLUXNET2015 sites, and AmeriFlux Base sites in an XLSX format. These files contain data such as CO_2_ concentration, temperature, and humidity. Data for Figures 1–6, S1, S2, S3, S4, and S5 can be found in these XLSX files. Additionally, optimized productivity, cost, and corresponding adsorption and desorption time data for all months at SPRUCE site 07 are available in an XLSX file. Data for Figures 7–10 are included in that file.

## Supporting Information Available

Additional data and results (PDF)
