# Supplementary material for: Incorporating Diurnal and Meter-Scale Variations of Ambient CO2 Concentrations in Development of Direct Air Capture Technologies
Source: ACS Sustain Chem Eng. 2024 Oct 25;12(45):16680–91. doi: 10.1021/acssuschemeng.4c06158 (PMC11558671; doi:10.1021/acssuschemeng.4c06158)
Supplement: Supplementary file 1 — sc4c06158_si_001.pdf [file sc4c06158_si_001.pdf]

Incorporating Diurnal and Meter-scale Variations of Ambient CO_2 Concentrations in Development of Direct Air Capture Technologies

Shubham Jamdade[1], Xuqing Cai[1], Melissa R. Allen-Dumas[2], and David S. Sholl[2]

[1]School of Chemical & Biomolecular Engineering, Georgia Institute of Technology, Atlanta, Georgia 30332-0100, United States
[2]Oak Ridge National Laboratory, Oak Ridge, TN 37830, United States

*Corresponding author email: shollds@ornl.gov

I. Variations in ambient conditions on vertical scales of meters 2

II. FLUXNET2015 site details and AmeriFlux BASE data source 4

III. CO_2 level variations with respect to tower height at selected FLUXNET2015 sites 6

The ZIP file with all the data files is available at this LINK. The ZIP file includes hourly atmospheric condition data from SPRUCE site 07, FLUXNET2015 sites, and AmeriFlux Base sites in an XLSX format. This file contains data such as CO_2 concentration, temperature, and humidity. Data for Figures 1, 2, 3, 4, 5, 6, S1, S2, S3, S4, and S5 can be found in these XLSX files. Additionally, optimized productivity, cost, and corresponding adsorption and desorption time data for all months at SPRUCE site 07 are available in an XLSX file. Data for Figures 7, 8, 9, and 10 are included in these files.

I. Variations in Ambient Conditions on Vertical Scales of Meters

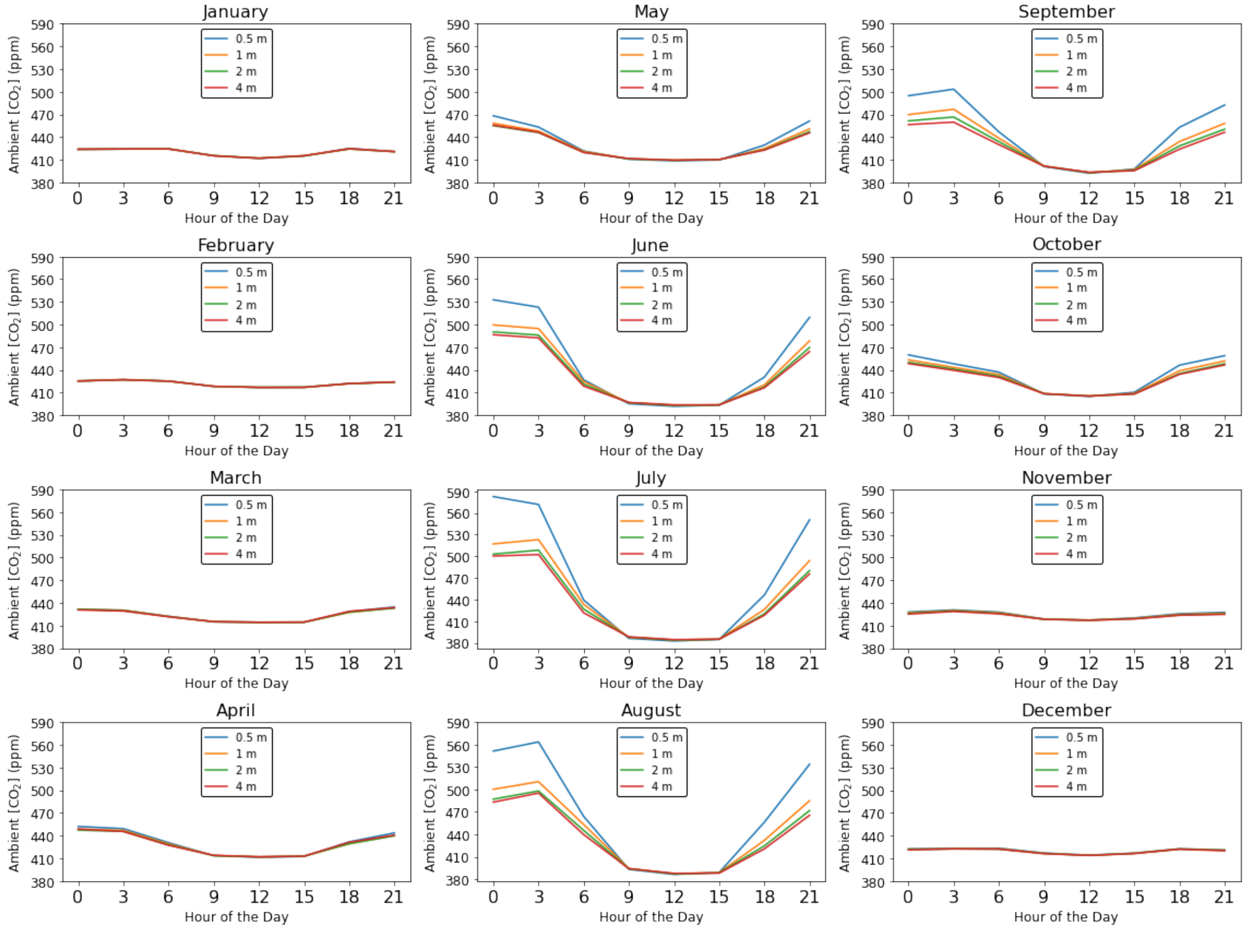

Figure S1: The average ambient CO_2 concentrations at a height of 0.5, 1, 2 and 4 meters from SPRUCE site 07 plotted at three-hour intervals for all the months in 2017. The CO_2 concentration value at each specified hour is the average concentration over a three-hour period starting from that hour averaged across all days of the month. The same vertical axis is used in each plot.

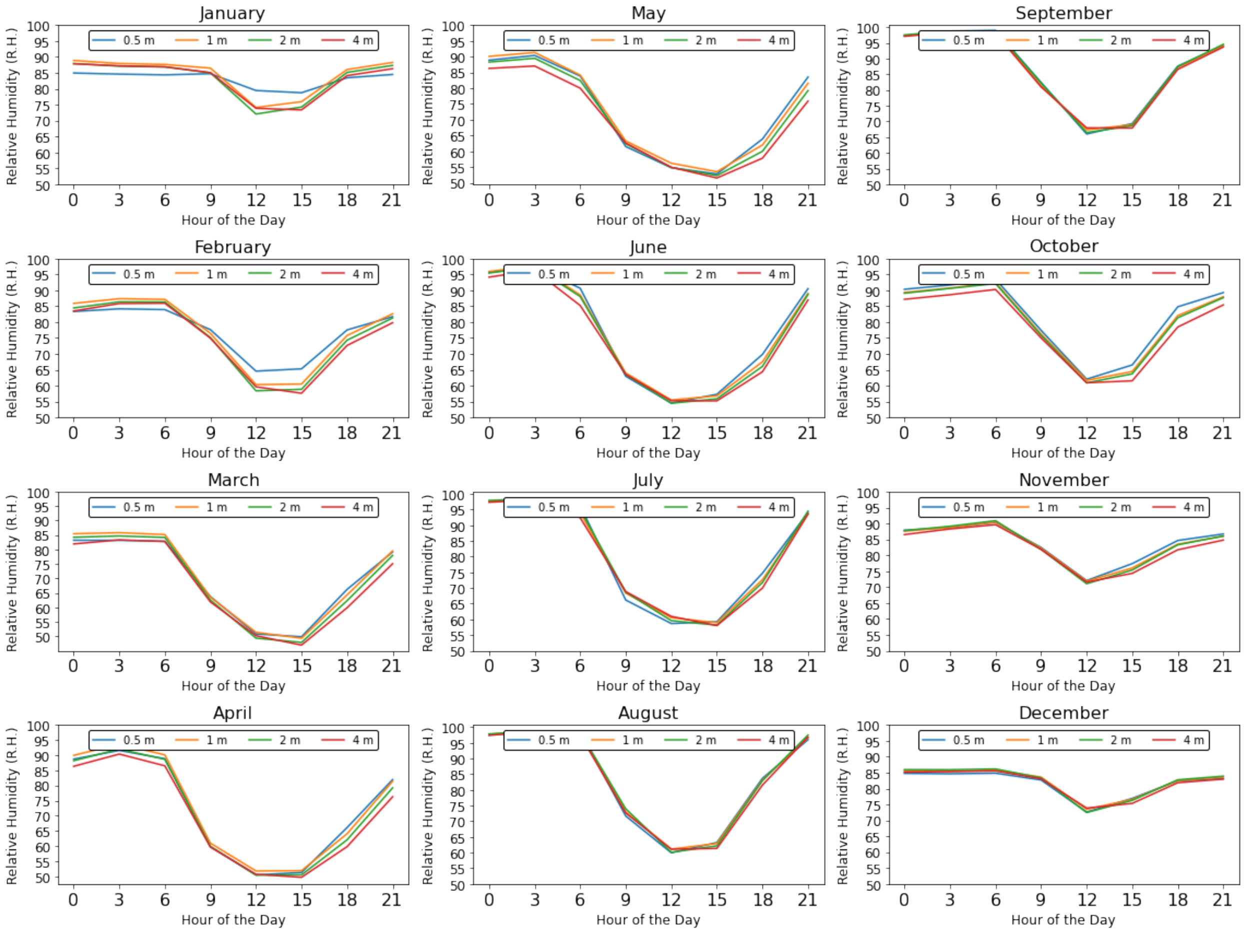

Figure S2: The average ambient relative humidity at a height of 0.5, 1, 2 and 4 meters from SPRUCE site 07 plotted at three-hour intervals for all the months in 2017. The relative humidity value at each specified hour is the average concentration over a three-hour period starting from that hour averaged across all days of the month. The same vertical axis is used in each plot.

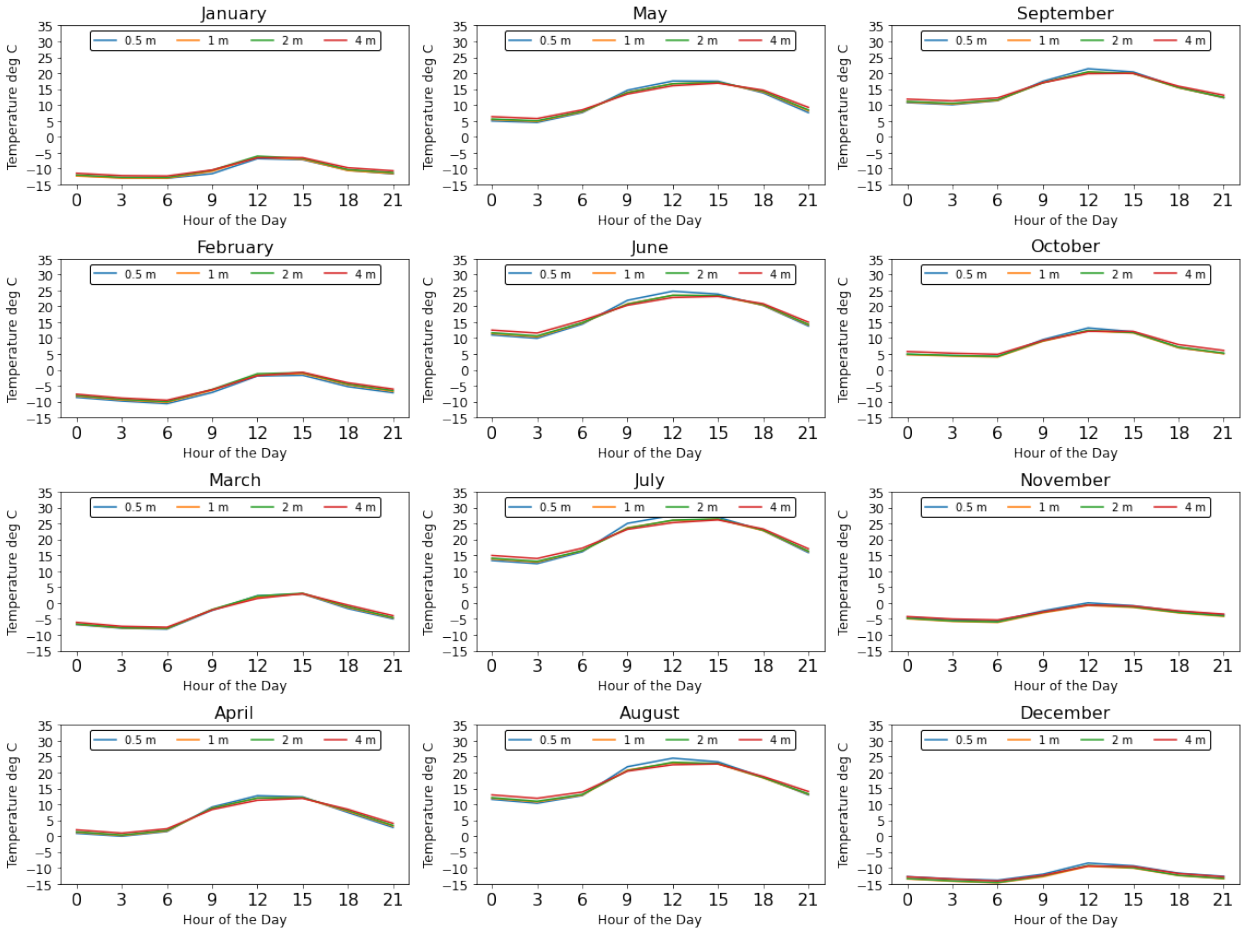

Figure S3: The average temperature at a height of 0.5, 1, 2 and 4 meters from SPRUCE site 07 plotted at three-hour intervals for all the months in 2017. The temperature value at each specified hour is the average concentration over a three-hour period starting from that hour averaged across all days of the month. The same vertical axis is used in each plot.

II. Sites details for selected sites from FLUXNET2015

FLUXNET2015 data source: https://fluxnet.org/data/download-data/
To download the data files specific to the below sites, download either of SUBSET or FULLSET Data Product by entering the corresponding Site ID
Table 1: Details for sites selected in this study from FLUXNET2015 dataset

Region	Site ID	Location Elevation (m)	Tower height (m)	Total height (m)	Years of data availability
Wisconsin	US-Syv	540	36	576	2001-2014
Wisconsin	US-WCr	520	29.6	550	1999-2014
Wisconsin	US-Los	480	10.2	490	2000-2014
Wisconsin	US-PFa	470	30	500	1995-2014

Toronto	CA-TP1	265	2	267	2002-2014
Toronto	CA-TP3	184	16	200	2002-2014
Toronto	CA-TP4	184	28	212	2002-2014
Toronto	CA-TPD	260	35.7	296	2012-2014
Arizona	US-SRG	1291	3.25	1294	2008-2014
Arizona	US-SRM	1120	7.82	1128	2004-2014
Michigan	US-Oho	230	32	262	2004-2013
Michigan	US-WPT	175	2.7	177.7	2011-2013
Michigan	US-CRT	180	2	182	2011-2013
California	US-Ton	177	23.5	200.5	2001-2014
California	US-Var	129	2	131	2000-2014

AmeriFlux BASE data source: https://ameriflux.lbl.gov/data/download-data/
Download the data files by entering the corresponding Site ID (for example: US-MBP)

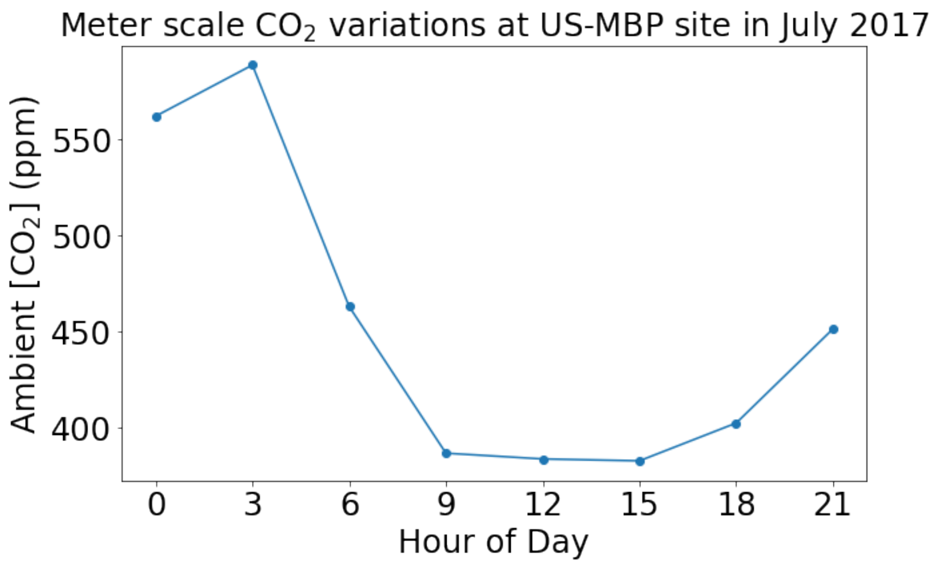

Figure S4: The average ambient CO_2 concentrations in July 2017 from site US-MBP in Minnesota plotted at three-hour intervals. The CO_2 concentration value at each specified hour is the average concentration over a three-hour period starting from that hour averaged across all days of the month.

III. CO_2 level variations with respect to tower height at selected FLUXNET2015 sites

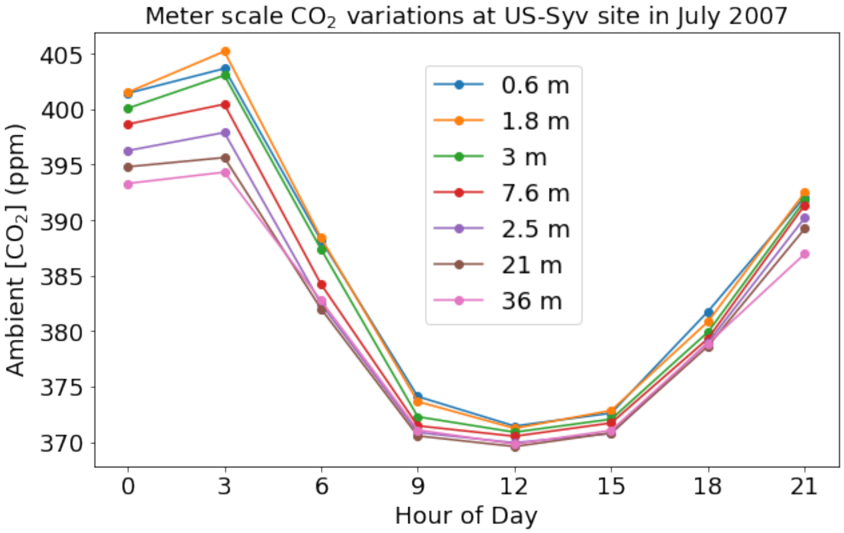

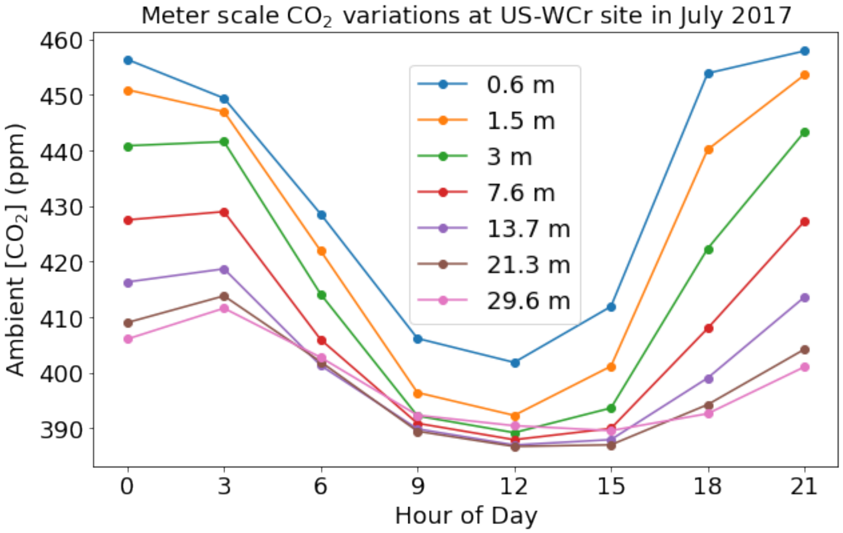

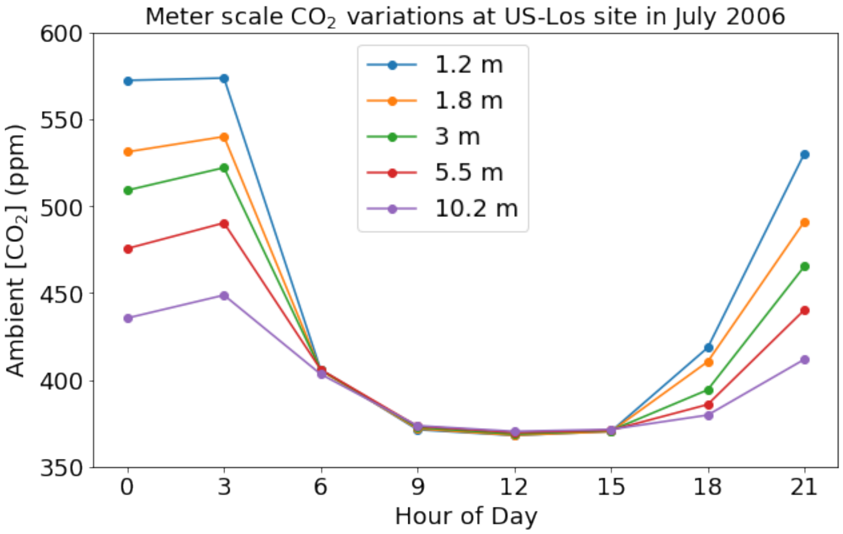

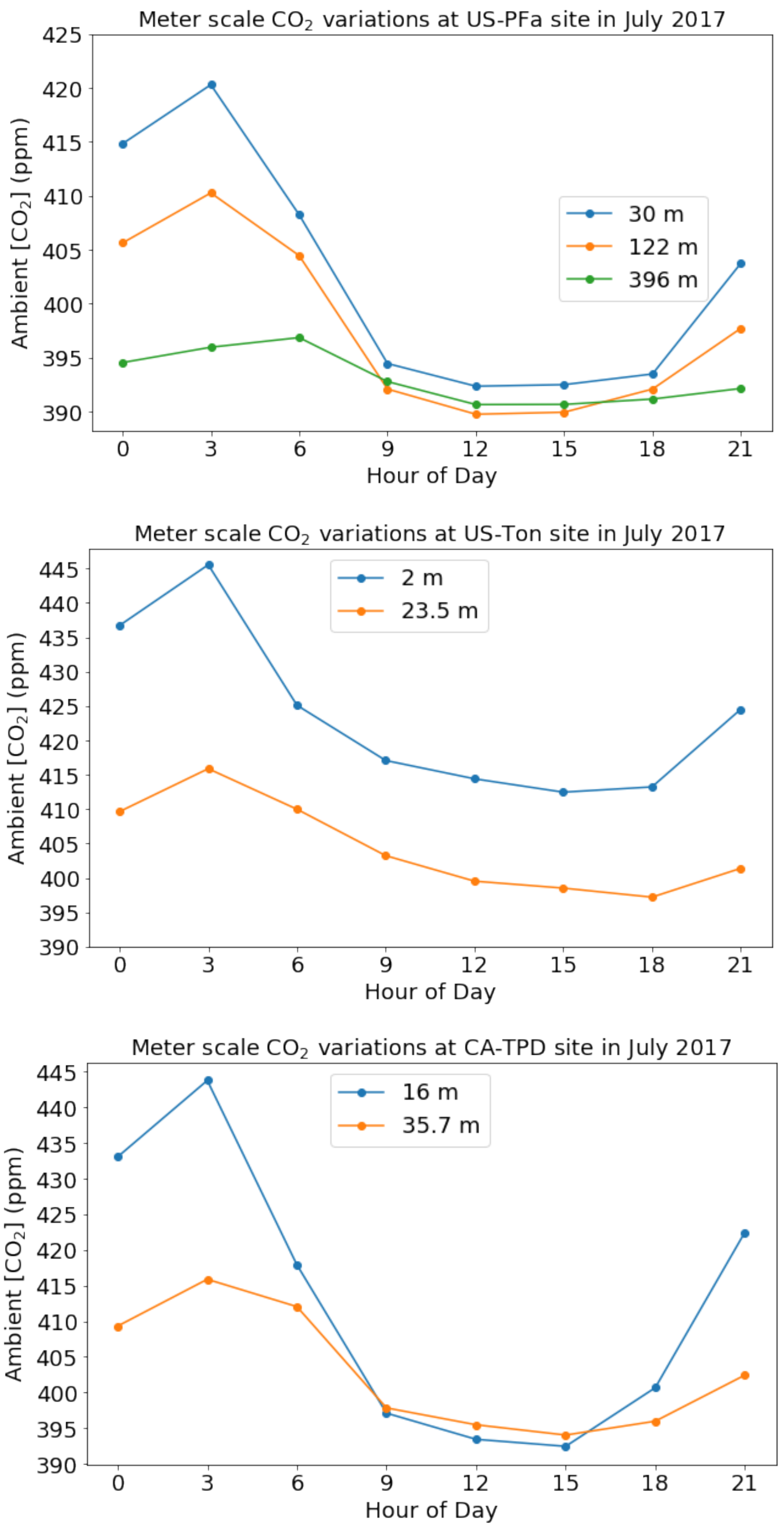

Figure S5: The average ambient CO_2 concentrations measured at various tower heights at the respective site in the month of July plotted at three-hour intervals. The legend in the plots represents the tower height. The CO_2 concentration value at each specified hour is the average concentration over a three-hour period starting from that hour averaged across all days of the month.
